# Stakeholder perceptions on scaling-up community-led interventions for prevention and control of non-communicable diseases in Bangladesh: a qualitative study

**DOI:** 10.1186/s12889-023-15551-9

**Published:** 2023-04-20

**Authors:** Kohenour Akter, Abdul Kuddus, Tasnova Jeny, Tasmin Nahar, Sanjit Shaha, Naveed Ahmed, Carina King, Malini Pires, Hassan Haghparast-Bidgoli, Kishwar Azad, Edward Fottrell, Joanna Morrison

**Affiliations:** 1 Diabetic Association of Bangladesh - Centre for Health Research and Implementation (BADAS-CHRI), BIRDEM, Ramna, Dhaka, 1000 Bangladesh; 2 Karolinska Institutet, K9 Global Folkhälsa, K9 GPH Stålsby Lundborg Alfvén, Stockholm, 171 77 Sweden; 3 UCL Institute for Global Health, 30 Guilford Street, London, WC1N 1EH UK

**Keywords:** Scale-up, Type 2 diabetes, Bangladesh, Sustainability, Policy, Health systems

## Abstract

**Background:**

Engaging communities is an important component of multisectoral action to address the growing burden of non-communicable diseases (NCDs) in low- and middle-income countries. We conducted research with non-communicable disease stakeholders in Bangladesh to understand how a community-led intervention which was shown to reduce the incidence of type 2 diabetes in rural Bangladesh could be scaled-up.

**Methods:**

We purposively sampled any actor who could have an interest in the intervention, or that could affect or be affected by the intervention. We interviewed central level stakeholders from donor agencies, national health policy levels, public, non-governmental, and research sectors to identify scale-up mechanisms. We interviewed community health workers, policy makers, and non-governmental stakeholders, to explore the feasibility and acceptability of implementing the suggested mechanisms. We discussed scale-up options in focus groups with community members who had attended a community-led intervention. We iteratively developed our data collection tools based on our analysis and re-interviewed some participants. We analysed the data deductively using a stakeholder analysis framework, and inductively from codes identified in the data.

**Results:**

Despite interest in addressing NCDs, there was a lack of a clear community engagement strategy at the government level, and most interventions have been implemented by non-governmental organisations. Many felt the Ministry of Health and Family Welfare should lead on community engagement, and NCD screening and referral has been added to the responsibilities of community health workers and health volunteers. Yet there remains a focus on reproductive health and NCD diagnosis and referral instead of prevention at the community level. There is potential to engage health volunteers in community-led interventions, but their present focus on engaging women for reproductive health does not fit with community needs for NCD prevention.

**Conclusions:**

Research highlighted the need for a preventative community engagement strategy to address NCDs, and the potential to utilise existing cadres to scale-up community-led interventions. It will be important to work with key stakeholders to address gender issues and ensure flexibility and responsiveness to community concerns. We indicate areas for further implementation research to develop scaled-up models of community-led interventions to address NCDs.

## Background

Noncommunicable diseases (NCDs) such as heart disease, stroke, cancer, diabetes, chronic respiratory diseases, and mental illness, cause nearly three-quarters of global mortality, and 86% of deaths occur in low- and middle-income countries (LMICs) [1]. Prevention strategies such as promoting healthy diets, physical activity, reduced alcohol use and tobacco use cessation can reduce premature death and disability from NCDs. Preventing NCDs also reduces the risk of developing co-morbidities alongside existing illness. There is a need to develop the evidence base about how prevention and control of NCDs can be enabled at a population level in LMICs to prevent premature mortality.

The WHO recommends multi-sectoral approaches which include diverse partnerships with civil society and private sector entities to address NCDs. Objective three of the WHO global action plan 2013–2020 focuses on reducing modifiable risk factors for NCDs and underlying social determinants through creation of health promoting environments [2]. Engaging communities can be effective at creating enabling and health promoting environments [3], but there has been little research about how to effectively engage communities [4] and scalability needs to be considered [5]. Scalability of community engagement requires analysis of policy, health systems and community contexts, and stakeholder-engaged research. In 2019, an approach to engage communities—community groups using a participatory learning and action (PLA)—was tested through a cluster randomised controlled trial in rural Faridpur, Bangladesh, and showed a 64% relative reduction in the combined prevalence of diabetes and intermediate hyperglycaemia (an absolute reduction of 20.7%) [6]. The intervention also significantly increased knowledge about causes, symptoms, and complications of diabetes. Awareness of diabetes status also increased in PLA areas relative to control. We describe the PLA approach and analyse stakeholder perceptions of the issues affecting scale-up of community-led interventions, and specifically PLA in Bangladesh, to inform the development of future interventions.

### Participatory Learning and Action (PLA) community groups

Health promotion through community groups is a popular public health strategy. Groups which develop community capacity have a stronger evidence-base for health improvements in LMICs than other types of group interventions [7]. One such approach, where groups and communities are facilitated by lay community members to address locally defined problems through a community PLA cycle has had particular success in improving maternal and newborn survival [8]. Recent research from rural India has shown that the intervention remains effective at reducing neonatal mortality when implemented at-scale through front-line health workers [9]. We adapted this PLA intervention to address T2DM in rural Faridpur, Bangladesh and tested its’ effectiveness through a cluster randomised controlled trial. Local male and female facilitators organised community groups of men and women who discussed how to prevent and control type 2 diabetes and considered the barriers to enacting health promoting behaviours. Groups then prioritised barriers and suggested strategies to address these which were presented and discussed at larger community gatherings. Communities implemented strategies which addressed barriers to physical activity for women, they sought community-based blood glucose testing through village doctors, and made home visits to promote healthy eating and tobacco reduction [10, 11]. The intervention was effective in reducing the incidence and prevalence of T2DM amongst all wealth groups and was cost effective [6, 12]. Given the success of the intervention, further research is required to test the intervention at scale and explore with stakeholders how it could be effectively scaled-up and integrated into existing policies and plans.

### Bangladesh NCD policy

Bangladesh has a high NCD burden, and population-based surveys have shown that around a third of the population have T2DM or intermediate hyperglyaecmia [13]. The NCD multisectoral action plan recognises the importance of prevention through community engagement [14, 15] but implementation has been slow. Delays in budgetary release and regular transfer of civil servants have challenged its’ implementation [16]. In addition, the plan has been criticised for minimal engagement of non-health sector agencies [17] and a lack of power to enforce action [15].

### Government health services for NCDs in Bangladesh

Community health services are implemented through the directorate general of family planning which runs family welfare centres, and the directorate general of health services which runs community clinics (CCs). Community health services have, until recently, been focused on maternal and reproductive health. The 4^th^ Health Population and Nutrition Sector Development Programme 2017–2022 included screening of NCDs in the services that should be provided at the CC [18]. Health promotion, screening (such as blood glucose testing and blood pressure measurement), referral and management of NCDs has been added to CC responsibilities. Research shows that plans to promote a healthy lifestyle, screen for early diagnosis, and manage NCDs through community health facilities have not been well implemented [19–21]. A WHO evaluation found that only 31% of CCs reported screening for NCDs [18]. Human resource shortages, poor budget utilisation, a lack of effective referral mechanisms, weak governance and poor monitoring and surveillance have impeded implementation of community based NCD services [19–21].

In 2018/9 a government financed multi-purpose health volunteer (MHV) programme was piloted in 107 sub-districts (upazillas) of eight districts and extended to 88 upazillas in 2019/20 [22]. Women are prioritised for this community-based position who should conduct health promotion, referral of pregnant women, referral for NCD screening (including T2DM and hypertension), nutrition counselling, collection of population data and vital registration, and facilitate community group meetings and community support group meetings. One MHV should cover 250–300 households and is paid up to a maximum of 3600 BDT (35 USD) per month through performance-based incentives. There are five MHVs per CC. MHV are responsible to the Community Health Care Provider in the CC and to a committee at the upazilla health level which is chaired by the Upazilla Health and Family Planning Officer (UH&FPO). MHVs submit monthly reports through an online system, which can be viewed at the national, upazilla, and community level. A UNICEF report published in 2022 [23] suggested that a lack of coordination between MHV and other community health workers was problematic, and their supervision and monitoring needs to be clarified and strengthened.

### Non-government health services for NCDs in Bangladesh

Bangladesh has a pluralistic model of health care provision. The private sector provides curative and diagnostic services but does little to promote prevention of NCDs. Key non-governmental health care providers for NCDs are National Institute of Cardiovascular Disease (NICVD); the National Heart Foundation (NHF) and the Bangladesh Institute of Research and Rehabilitation for Diabetes (BIRDEM), part of the Diabetic Associations of Bangladesh (BADAS) [24]. BADAS is the second largest health provider in Bangladesh, and it has around 114 health care facilities and more than 500 accredited Diabetes Centres. These centres provide free or low-cost care and conduct occasional awareness raising activities [25].

## Methods

### Data collection

**Fig. 1:**
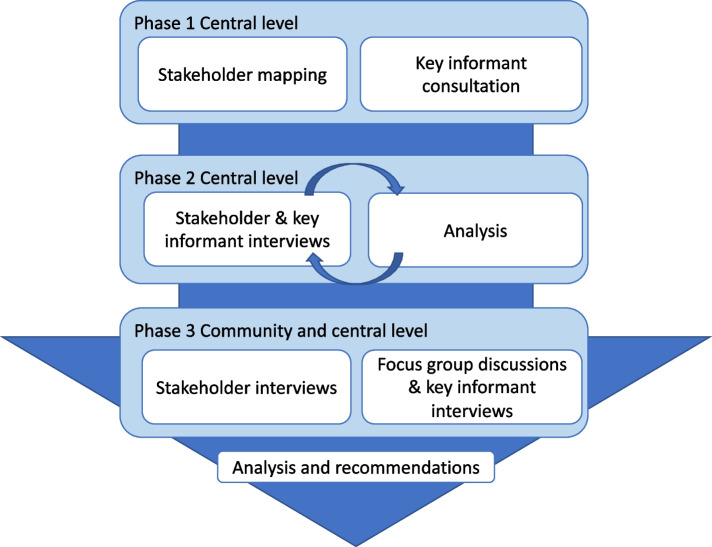
Data collection and analysis process

We collected data in three iterative phases informed by Schmeer’s stakeholder analysis guide [26] (Fig. 1). This guide is an established framework and has been used in stakeholder analysis in Bangladesh previously [17]. Data were collected in Dhaka, the capital city of Bangladesh, and in rural Faridpur, south central Bangladesh where a PLA intervention had been implemented from June, 2016, to December, 2017 [6]. Data collection was somewhat protracted because of the COVID-19 pandemic and data were collected between November 2019 and November 2021.

#### Phase one

The objective of phase one was to map stakeholders. The Diabetic Association of Bangladesh Centre for Health Research and Implementation (BADAS-CHRI) research team and JM brainstormed 18 stakeholders from different sectors (research, non-governmental organisation, Ministry of Health and Family Welfare, donor agencies). Stakeholders were defined as any actor who could have an interest in the intervention, or that could affect or be affected by the intervention. A trained qualitative researcher (KAk) then discussed this list with three key informants from research and non-governmental sectors who validated, added stakeholders, and helped prioritise stakeholders to interview (Table 1).

**Table 1:** Data collection with central and community level stakeholders

Type of organisation	Male	Female
Research	2	2
NGO	3	-
Ministry of Health & Family Welfare and health sector	3	1
Donor agency	1	-
**Total**	**9**	**3**

#### Phase two

The objective of phase two was to understand stakeholder views on how best to engage communities for NCD prevention and control and explore current NCD community engagement strategies. KAk interviewed five stakeholders and three stakeholders who were also consulted in phase one. Two semi-structured interviews (SSI) were online and six were face-to-face. We used topic guides that were developed following Schmeer guidance, adding questions specifically about how a PLA intervention could be scaled-up, considering the WHO health system building blocks of service delivery, health workforce, health information systems, access to essential medicine, financing and leadership governance [27]. JM and KAk discussed each interview after it was conducted and adapted the topic guide between interviews. This enabled us to explore ideas raised by previous participants. Two SSIs were conducted over several days because of stakeholder time limitations.

#### Phase three

The objective of phase three was to understand the feasibility and acceptability of three scale-up strategies which were developed from data in phase two. KAk interviewed two central level stakeholders from phase two and four community level stakeholders in Faridpur where we had implemented the PLA intervention. Topic guides explored how the different scale-up options could affect stakeholders, the feasibility of the options, and the structures or systems that would need to be engaged.

In addition, we sought community views on scale-up options. A community survey in Faridpur identified women’s and men’s groups that had and had not continued meeting after the PLA intervention was no longer supported by BADAS-CHRI [28]. We purposively sampled six groups based on gender and when the group had last met. Key informants – community members who lived nearby the group meeting place, but who hadn’t been group members—were located through community based BADAS-CHRI staff. A female qualitative researcher (TJ) conducted focus group discussions (FGDs) with men’s and women’s groups and key informant interviews (KII). Data were collected in five villages of four upazillas (Table 2). TJ used topic guides to discuss the feasibility and acceptability of options for scale-up.

**Table 2:** Data collection with community members who had participated in PLA groups

	**Women’s group**		**Men’s group**	
**Method**	**FGD**	**KII**	**FGD**	**KII**
Groups that had met	2	2	2	2
Groups that had not met	1	1	1	1
**Total**	**3**	**3**	**3**	**3**

### Data analysis

Data were collected in Bangla, recorded and summaries written in English by KAk and TJ. KAk and JM read, made notes and discussed the data after each phase of data collection. Our analysis method was informed by the critical paradigm [29]. We wrote and shared thick, rich descriptions of the data after the first and second phases and discussed these with the wider research team. This helped us to check our interpretations, extract themes for further exploration or confirmation and plan the next phase of data collection. The process of writing, reflecting, and discussing the data enhanced the rigour of our analysis, enabling researcher subjectivity to be made explicit and checked through team analysis. Our process of comparing our data with the Schmeer framework, developing themes and topics collaboratively and then exploring them with participants through an iterative approach enabled member-checking of interpretations with participants [29]. After data collection was complete, we deductively analysed data in Nvivo (version Release 1.7.1) using categories from stakeholder analysis literature (positionality, ideas, context, and issues) and inductively coded with additional categories identified in the data. For example, within the code ‘context’ we identified health systems context, policy context, and community engagement context as sub-themes.

## Results

First, we describe the important contextual factors which our analysis revealed. Then we describe perceptions about the PLA intervention research which would also affect scale-up. We then present our analysis of stakeholder opinions of different methods of scaling-up PLA, before discussing the implications of our results for scale-up of community-led interventions to address NCDs in LMICs.

### Policy context

Most stakeholders said that NCDs were a priority for the government, and the Ministry of Health and Family Welfare had made more progress in implementing the multisectoral action plan than other sectors. Political issues impeding implementation, such as frequent transfer of government officials, and a lack of leadership, were identified by government and non-governmental stakeholders:

“There was leadership at one point. The (government) did some work when (the multi-sectoral action plan) started, they made a guideline. But frequent leadership changes have had an impact. I don’t know the present situation of leadership or ownership” (SSI 09).

### Lack of a clear community engagement strategy

There wasn’t a clear strategy to engage with communities to prevent NCDs. Stakeholders mentioned ad hoc initiatives and collaborations between the government, NGOs and community-based organisations in selected parts of the country, but these were never discussed as part of a national or comprehensive strategy. Community groups were mentioned but their method of engagement with communities was unclear, and one stakeholder admitted:

“These meetings are not held in most areas” (SSI 05).

Government and non-government stakeholders indicated that collaborating with NGOs enabled implementation of NCD programmes, despite difficulties with sustainability.

### Health systems context

Government health services were overloaded and unable to cope with demand for NCD services:

“Other organizations should come forward as it is difficult for government to manage the huge burden of NCDs” (SSI 02).

Most national level stakeholders felt that human resources were available at the community level to implement interventions, but they needed to be better co-ordinated, supported and supervised to engage communities. But local level stakeholders noted that most community health workers were based in health institutions, and services would suffer if they worked in the community or supervised work in the community:

“The Health Assistant must give vaccinations for two days, then has to sit in the community clinic for three days and then she goes to the field for one day to invite for vaccination or do other jobs. Recently she has also started working on the COVID vaccine. So, if another burden is added then she would not be able to do it well” (SSI 013).

The MHV was a notable exception being based in the community, and many felt that her role was amenable to implementing community-led interventions.

Financial barriers to implementing community-led interventions were discussed, but central level stakeholders felt that these were not insurmountable as budget had been allocated for NCDs. Local level financing was suggested, for example:

“(The) Union health and education standing committee have some budget for village health programmes. If health programmes and local government can cooperate with each other, then health volunteers and health assistants …can facilitate these activities at a wider scale” (SSI 08).

Local level stakeholders discussed the need for full integration of an intervention into government systems if they were locally financed, but this would be difficult because of the pressure on existing human resources. A local government stakeholder said:

“With our existing staff it would be very difficult to do this as we only have 40% of posts filled. It is only feasible if we fill all the posts” (SSI 013).

### PLA for awareness raising

Most stakeholders understood that the intervention had been effective at reducing the incidence T2DM through increasing awareness and individual action to prevent NCDs, particularly among women and marginalised communities. They felt it was important to raise awareness in communities:

“To reach huge numbers of people we need prevention through health education. For health education, we need to develop messages considering the disease and community. So, for health education involvement of the community is very important” (SSI 01).

Awareness raising for prevention was perceived to be an important component of the multisectoral action plan by government and non-government stakeholders, and most understood the mechanism of the PLA intervention in this way.

### PLA for community action

The community action part of the PLA intervention was not well understood, and it was more difficult for KAk to explain. When we discussed the strategies undertaken by communities, such as village doctors doing blood glucose tests, stakeholders (particularly those working for the government health sector) were concerned. Village doctors are largely unregulated and often unqualified, and stakeholders were sceptical of the quality of care that would be received. They were concerned about unsafe prescribing which could unnecessarily increase out-of-pocket expenses:

“I am disagreeing with your programme in one part as I personally discourage doing a test for diabetes with the village doctor for several reasons: village doctors have the tendency to give treatment and they have started to maltreat. They start to prescribe unnecessary medicine or medicine which is costly or that which should be prescribed later. This is a costly burden for people with diabetes” (SSI 013).

Stakeholders may have also been concerned about endorsing an intervention which could be interpreted to promote the use of village doctors.

Most stakeholders felt that community engagement should be managed through the health sector and be linked with treatment services:

“I think this is the responsibility of health sector. As this is a health-related problem, so the doctors and Ministry of Health and Family Welfare have the first and foremost responsibility” (SSI 10).

The engagement of doctors and/or health workers was thought to be necessary for the success of the intervention, and most felt that it should be implemented as a stand-alone intervention linked to the health sector. Almost all stakeholders couldn’t see any benefits to involving pharmaceutical companies who would be uninterested without a direct link to increased prescribing.

### Scale-up options

#### Implementation of the intervention through BADAS

Several participants suggested that BADAS was well placed to implement the intervention, as they had been doing so, and had nation-wide reach:

“BADAS can play huge role to scaling up as BADAS has more than 100 hospitals in all over Bangladesh” (SSI 02).

They were also a trusted organisation that was generally perceived to provide good quality care. Stakeholders discussed other awareness raising interventions that had been run in partnership with the government of Bangladesh as examples of how this could be done. But staff at BADAS were more sceptical. Almost all BADAS programmes are curative, not preventative and most of the staff are institution based:

“We don’t go to the community, the community comes to us” (SSI 011).

Doctors are perhaps not the most appropriate cadre to be conducting community mobilisation, and they may be unwilling or lack the skills necessary to facilitate discussion. BADAS lacks the implementation and supervision structures for community-based workers and would require specific budget to implement PLA at scale.

#### Self-sustaining community groups

We discussed whether group members would pay the facilitator for their work. Stakeholders told us that group members would not pay for awareness raising activities and would be more likely to expect an incentive to join a group. Merging PLA groups with other groups, such as savings and credit groups could be beneficial because such groups were widespread, with a functioning supervision structure. But many felt that integrating two different topics would be challenging:

“If you merge your group with micro-credit organisation then it wouldn’t help, I think. The aim of a micro-credit organisation is different. They deal with money. The awareness raising discussion wouldn’t be given importance” (KII 020,401).

We discussed whether PLA facilitators could charge money for blood glucose testing services and running community groups. This was a popular idea as this would bring services closer to communities. Community members emphasised the need for good quality testing equipment, training, and supervision so the facilitator would be trusted:

“The machines must work properly. Some machines don’t give a correct result, and then people would just be spending money but would not get any benefits” (FGD Men 1010501).

#### Implementation by multipurpose health volunteers (MHV)

Health workers running PLA groups was also a popular option among community stakeholders. People would be motivated to attend and hear from a health expert. They emphasised supervision and support to ensure they worked according to expectations:

“If the health worker is not dedicated then it won’t work. They must be dedicated and attend regularly” (KII 1020101).

Some stakeholders suggested implementing the intervention through MHVs, but a few felt the MHV programme had not been effective:

“I have heard some volunteers are working in some areas -though I cannot remember now- but I don’t think that they are working well” (SSI 010).

Others said that MHVs had been focused on maternal health issues:

“The main point is, the volunteer will supervise all the pregnant women in their area” (SSI 013).

Their role in running community groups was discussed as a good integration point for community-led interventions, but MHVs have had difficulties engaging men in discussion:

“Men are not available and if anyone wants to involve them, they need to involve them at their convenient time. Government health workers, community groups and MHVs have found it difficult to involve men because of this” (SSI 04).

## Discussion

In order to implement effective population-based interventions to address NCDs at scale, it is important to understand how context could affect scale-up [30]. Rigorous effectiveness research alone does not shift policy and programming and understanding contexts and systems, and the positionality of actors needs to be considered [31]. COVID-19 restraints affected our ability to meet with a broad range of stakeholders, and stakeholders often had limited time, but nevertheless, our iterative qualitative research with national and community-based stakeholders in Bangladesh has identified scale-up options, and factors affecting the success of these. The WHO suggests that innovations are more likely to be scaled-up if they have the CORRECT attributes [32]: **C**redibility, **O**bservable results; are **R**elevant and addresses key community concerns; provide a **R**elative advantage over existing practice; are **E**asy to implement; are **C**ompatible with established norms, values and existing programmes; and are **T**estable. We have assessed a community-led PLA intervention against these criteria based on our data (Table 3) and discuss compatibility issues that are important for the context of Bangladesh.

**Table 3:** Analysis of attributes which enhance scalability of innovations(World Health Organization, 2009) [32]

Intervention attributes	Community-led intervention
Credible and has observable results	• Research about the effectiveness of PLA was considered robust, and the intervention effectiveness was believable • Awareness raising was perceived an important way to change behaviours
Relevant and addresses key community concerns	• Addressing NCDs was on the policy agenda, but community engagement had not been given much attention by donors or government • NCDs were of key concern to communities
Provides a relative advantage over existing practice	• Community engagement approaches have been implemented in a fragmented and localised way and a systematic approach was perceived to be an improvement • Community engagement approaches were perceived as similar in method and content and PLA was only considered a distinct and preferable intervention by a few stakeholders
Easy to install	• Using community groups as a method of engaging the community was not perceived as difficult to implement • Stakeholders said that it would be challenging to fit the PLA intervention within the existing government health system and felt that the intervention was easier aligned with an NGO implementation modality
Compatible with established norms, values, and existing programmes	• Stakeholders felt that the intervention should be implemented through the health sector, but the need to reach both men and women and the lack of community-based (as opposed to facility-based) health workers make this approach problematic • Stakeholders had difficulty reconciling the community-led approach with the need to promote ‘best practice’ • MHVs offer a potential scale-up modality, but adaptions to their role would be necessary
Testable	• A PLA intervention focused on addressing T2DM has been tested through a cluster randomised controlled trial, and stakeholders perceived the intervention to be testable by a research organisation • The need to monitor and supervise community engagement interventions was emphasised by stakeholders

World Health Organization. (2009) [32]. Practical guidance for scaling up health service innovations. World Health Organization

### Gender issues

PLA groups were split into men’s and women’s groups because of gender and cultural norms which prevent men and women from mixing freely, and often mean that they are available at different times of the day. For example, men often preferred to meet in the evening, and women preferred to meet during the day. This flexibility has been a key success component of PLA [6, 33] and has enabled engagement and improved health outcomes among men and women, in a range of age and wealth groups [12]. This flexibility might be difficult for a government or NGO worker with strict working hours. Safety and reputational risk is of concern for women moving around during quiet times [34], and therefore a female facilitator like an MHV might find it challenging to run men’s groups in the evenings. A scaled-up approach would need to address these gender issues.

### Acceptability of community-led action

Whilst awareness raising activities through groups in communities was compatible with previous interventions, community-led action to problem solve was unfamiliar. PLA works through community prioritised action [10], and one such action was disagreeable to stakeholders: unqualified village doctors making home visits [35, 36]. Receipt of services close to home was particularly important to women whose movement was restricted due to purdah (seclusion) norms [37]. If communities sought actions which were not considered ‘best practice’ community action could be curtailed if scaled-up through government, which may limit the success of the intervention. Or, there could be potential for community-led interventions implemented through government systems to communicate community demands, such as a need for improved outreach facilities, directly to policy makers [38].

### NGO implementation and sustainability

Implementation of community-led approaches through NGOs may allow for flexibility in implementation, and this option was acceptable and familiar to stakeholders. The engagement of non-governmental actors has contributed to rapid improvements in health and development outcomes in Bangladesh [39], but NGOs require funding to implement programmes, which limit their sustainability. In Bangladesh, sustainability of NGO interventions is often addressed through charging for services, but some researchers have raised ethical concerns over those most marginalised being expected to ‘pay for their own development’ [40]. Piloting of this approach is necessary to understand equity concerns.

### Integrating reproductive health and prevention of NCDs

Nair et al. found that in India, the government saw the scale-up of PLA through ASHAs (community health workers) as an opportunity to develop their capacity and address multiple programmes at once [9], but this requires effective integration. It may take time for communities to trust a reproductive health focused community-based health worker/volunteer as a source of information and advice about NCDs, as has been shown in research from Uganda [41, 42]. Systematic reviews of community health workers in LMICs have found the need to supervise, remunerate, enable tracking of patient data, enable them to work autonomously, and ensure timely re-supply of medication and supplies to be important [43, 44]. Community Health Workers addressing NCDs in Uganda [45], Nepal [46], and India, have had some success although remuneration and supervision were challenging [47]. Further engagement to pilot PLA through MHVs may be a good opportunity to better integrate NCDs and reproductive health in government health systems.

### Problem framing

Whilst all stakeholders acknowledged that prevention was important, we noted a biomedical focus on control and management of T2DM. There was a focus on government health service screening and referral as opposed to prevention through engaging with cultural, environmental, or structural drivers of behaviours that increase risk of NCDs. The importance of NCD problem framing for multi-sectoral and community action has also been documented elsewhere [48, 49] and may be a persistent challenge to effective advocacy for community-led, preventative approaches.

## Conclusion

A key component of a multisectoral approach to NCDs is community engagement, and one intervention that shows promise is a community-led intervention to prevent and control T2DM in rural Bangladesh. Our research found opportunities to scale-up this intervention through government and non-government sectors which require further implementation research to assess their cost, feasibility, acceptability, and equity. It will be important to advocate for prevention as an integral part of the multisectoral action plan and address issues of gender of implementers to ensure that men and women are equally reached through the scale-up mechanism. Fidelity to the flexible approach of PLA will be important to safe-guard to ensure that interventions are community-led and fit with the needs of communities.

## Data Availability

The datasets used and/or analysed during the current study are available from the corresponding author on reasonable request.

## Abbreviations

BADAS: Diabetic Associations of Bangladesh
BADAS-CHRI: Diabetic Association of Bangladesh Centre for Health Research and Implementation
BIRDEM: Bangladesh Institute of Research and Rehabilitation for Diabetes
CCs: Community Clinics
FGD: Focus group discussion
KII: Key informant interview
LMICs: Low-and middle-income countries
MHV: Multi-purpose health volunteer
NCD: Non-communicable diseases
NHF: National Heart Foundation
NICVD: National Institute of Cardiovascular Disease
PLA: Participatory learning and action
SSI: Semi-structured interview
T2DM: Type 2 Diabetes
UH&FPO: Upazilla Health and Family Planning Officer
USD: United States Dollar
WHO: World Health Organization
